# Magnetic Resonance Imaging of Intramyocardial Fat Deposition in Tuberous Sclerosis

**DOI:** 10.3390/diagnostics10121031

**Published:** 2020-12-01

**Authors:** Zoi Tsoumani, Melanie Greaves, Matthias Schmitt, Gaetano Nucifora

**Affiliations:** 1 Cardiac Imaging Unit, Wythenshawe Hospital, Manchester University NHS Foundation Trust, Manchester M23 9LT, UK; ztsoumani@yahoo.gr (Z.T.); melanie.greaves@mft.nhs.uk (M.G.); matthias.schmitt@mft.nhs.uk (M.S.); 2 Division of Cardiovascular Sciences, School of Medical Sciences, Faculty of Biology, Medicine and Health, Manchester Academic Health Science Centre, University of Manchester, Manchester M13 9PL, UK

**Keywords:** cardiac magnetic resonance, intramyocardial fat, tuberous sclerosis

## Abstract

Tuberous sclerosis complex (TSC) is a rare autosomal dominant neurocutaneous syndrome. The phenotype is highly variable and may affect several organ systems, the hallmark of the disease being widespread hamartomas or abnormal growth of normal tissues. Although cardiac rhabdomyomas are the most common cardiac manifestation of TSC, being developed quite early, even during the second semester of the gestation, they tend to regress spontaneously over a period of months or years. On the other hand, the presence of intramyocardial fat deposition has been significantly associated with brain involvement and other extracardiac manifestations. We report the case of a 37-year-old man with TSC who presented to hospital with loss of consciousness, head injury and amnesia and in whom cardiac magnetic resonance imaging revealed the presence of multiple areas intramyocardial fat deposition.

A 37 years-old man presented to our hospital with loss of consciousness, head injury and amnesia. The patient had been diagnosed with tuberous sclerosis (TS) due to mutation in gene TSC2 (encoding tuberin) at the age of two years. Manifestations of the disease in this patient included facial angiofibromas (Adenoma sebaceum), large bilateral renal angiomyolipomas, subependymal giant cell astrocytomas resected 20 and 2 years earlier, focal epilepsy, patches of retinal pigment epithelial hypopigmentation and retinal hamartomas.

His physical examination at time of hospital admission was unremarkable. Computed tomography of the brain did not show evidence of acute haemorrhage or recent segmental infarct but only evidence of previous craniotomy. His 12-lead electrocardiogram (ECG) and in-hospital telemetry ECG monitoring were normal. As the patient was known to have a cardiac rhabdomyoma at a younger age, an echocardiogram was performed, ruling out the presence of gross abnormalities and inflow/outflow obstruction. Cardiac magnetic resonance imaging was also performed for more detailed anatomical and structural assessment; this demonstrated normal cardiac anatomy with mild basal septal bulge and preserved biventricular function. There was no evidence of rhabdomyoma or thrombus. However, areas of intramyocardial fat deposition in the mid anterolateral left ventricle wall were seen, as well as minor fat deposition in the anterior wall (Figure 1). Intramyocardial fat deposition reveals high signal in b-steady-state free precession cine sequence-bSSFP (arrow in Figure 1A, mid short-axis view). This sequence provides fast cine imaging with high contrast between myocardium and blood and with tissue contrast depending on the T2/T1 ratio; consequently, myocardium exhibits intermediate signal intensity, while fat and free water (blood pool) high signal intensity [1,2]. The presence of intramyocardial fat was then confirmed by dedicated fat imaging magnetic resonance sequences. High signal areas were indeed documented in T1-weighted black-blood Turbo Spin Echo (TSE) sequence (arrow in Figure 1B, mid short-axis view and arrows in Figure 1C, 2-chamber view), corresponding to low signal in the T1-weighted black-blood TSE with fat suppression sequence (arrow in Figure 1D, mid short-axis view); these areas were hyper-enhanced in the contrast-enhanced inversion-recovery T1-weighted fast gradient-echo images obtained 10 min after injection of Gadolinium chelate contrast agent (i.e., late gadolinium enhancement technique; arrow in Figure 1E, mid short-axis view and arrows in Figure 1F, 2-chamber view). As a clear explanation of the initial clinical presentation (i.e., arrhythmias-related vs. neurological-related syncope) was not achieved, implantation of loop recorder for prolonged ECG monitoring was then planned.

Tuberous sclerosis complex (TSC) is a rare autosomal dominant neurocutaneous syndrome (incidence of approximately 1/6000 live births) caused by mutations in the genes TSC1 and TSC2, which encode hamartin and tuberlin. The phenotype is highly variable and may affect several organ systems (mainly the brain, kidneys, skin, lungs, liver, eyes, and heart), the hallmark of the disease being widespread hamartomas or abnormal growth of normal tissues [3].

Although cardiac rhabdomyomas are the most common cardiac manifestation of TSC, being developed quite early, even during the second semester of the gestation, they tend to regress spontaneously over a period of months or years [3]. More rarely, cardiac rhabdomyomas develops in the absence of TSC [4]. On the other hand, the presence of intramyocardial fat deposition has been significantly associated with brain involvement, as in our patient, as well as with other extracardiac manifestations [5,6,7]. Adriansen et al. reviewed the abdominal computed tomography scans that included at least the basal portions of the heart performed in 55 patients with TSC. Foci of fat attenuation within the myocardium were identified in 64% of the patients; they were well circumscribed and located in the interventricular septum, left ventricle wall, right ventricle wall, and papillary muscles [5]. Similarly, Shaaya and colleagues reviewed the chest CT scans of 73 individuals with TSC; myocardial fat-containing lesions were documented in approximately one-third of adolescents and adults with TSC. No difference was observed between the genders and the different types of mutation. Of note, compared to those patients without myocardial fat-containing lesions, those with cardiac fat were more than twice as likely to have an abdominal manifestation of TSC [6]. More recently, Tresoldi et al. reviewed the chest CT scans of 48 patients with TSC; one or more intramyocardial fat depositions was detected in 50% of subjects; importantly, the presence of intramyocardial fat deposition was significantly associated with brain and multiorgan involvement and the number of intramyocardial fat depositions per patient was associated with the degree of multiorgan involvement [7]. Of note, post-mortem analysis of the heart of patients with TSC showed focal areas of mature fat cells in the myocardium without associated inflammation, fibrosis, or entrapped myocardial cells and without a capsule [8].

The differential diagnosis of intramyocardial fat deposition typically includes various pathologic conditions, such as post myocardial infarction lipomatous metaplasia, arrhythmogenic cardiomyopathy, dilated cardiomyopathy, and muscular dystrophy [9,10]. All of these were able to be excluded based on individual patient’s history, clinical data, and cardiac magnetic resonance imaging results. In particular, cardiac magnetic resonance imaging should be always considered in the work-up of patients with TSC, due to its unique ability in providing tissue characterization and detailed anatomical and structural assessment of the heart.

## Figures and Tables

**Figure 1:**
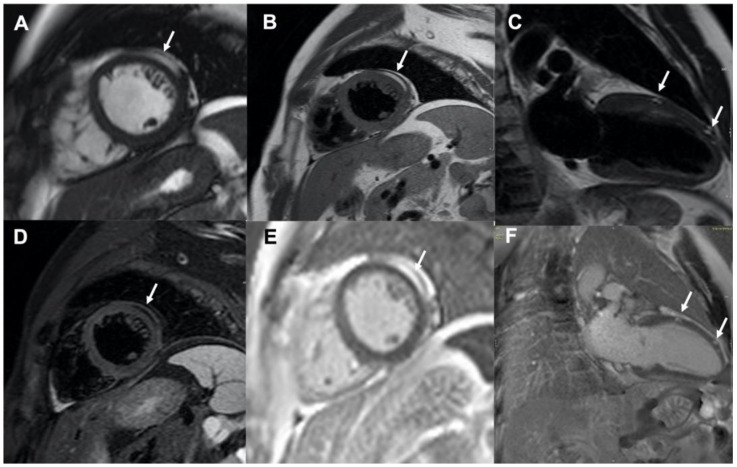
Cardiac magnetic resonance imaging showing multiple areas of intramyocardial fat deposition (white arrows); (**panel A**): b-steady-state free precession cine sequence, mid short-axis view; (**panels B,C**): T1-weighted black-blood Turbo Spin Echo images, mid short-axis view and 2-chamber view, respectively; (**panel D**): T1-weighted black-blood Turbo Spin Echo image with fat suppression, mid short-axis view; (**panels E,F**): late gadolinium enhancement images, mid short-axis view and 2-chamber view, respectively.
